# Phytochemical, Nutritional and Mineral Content of Four Edible Flowers

**DOI:** 10.3390/foods13060939

**Published:** 2024-03-20

**Authors:** Ilaria Marchioni, Morena Gabriele, Giulia Carmassi, Barbara Ruffoni, Luisa Pistelli, Laura Pistelli, Basma Najar

**Affiliations:** 1 Department of Food and Drug, University of Parma, Parco Area delle Scienze, 27/A, 43124 Parma, Italy; 2 Institute of Agricultural Biology and Biotechnology (IBBA), National Research Council (CNR), Via Moruzzi 1, 56124 Pisa, Italy; morena.gabriele@cnr.it; 3 Department of Agriculture, Food and Environment (DAFE), University of Pisa, Via del Borghetto 80, 56124 Pisa, Italy; giulia.carmassi@unipi.it (G.C.); laura.pistelli@unipi.it (L.P.); 4 Research Center for Vegetable and Ornamental Crops (CREA), Corso degli Inglesi 508, 18038 Sanremo, Italy; barbara.ruffoni@crea.gov.it; 5 Department of Pharmacy, University of Pisa, Via Bonanno 6, 56126 Pisa, Italy; luisa.pistelli@unipi.it; 6 Interdepartmental Research Center Nutraceuticals and Foods for Health (NUTRAFOOD), University of Pisa, Via del Borghetto 80, 56124 Pisa, Italy; 7 Pharmacognosy, Bioanalysis & Drug Discovery Unit, Faculty of Pharmacy, Free University of Brusseles, Bld Triomphe, Campus Plaine, CP 205/9, B-1050 Bruxelles, Belgium; basma.najar@ulb.be

**Keywords:** aroma profile, vitamin B quantification, mineral content, polyphenols, carotenoids, soluble sugars, *Fuchsia*, *Dianthus*, *Viola*, *Cucurbita*

## Abstract

The growing interest in functional foods is driven by the exploration of new foods with positive health effects. Pleasant sensory features are essential for consumer acceptance. In this work, we investigated the composition of the bioactive compounds, antioxidant activity, and aroma profiles of four edible flowers: *Cucurbita moschata* Duchesne, *Dianthus chinensis* L., *Fuchsia regia* (Vand. ex Vell.) Munz., and *Viola cornuta* L. For the first time, we quantified the water-soluble group of B vitamins. Significant variations in the content of soluble sugars, vitamins, and secondary metabolites were observed. *V. cornuta* showed the highest concentration of vitamin C and carotenoids, while *C. moschata* had the highest content of vitamin B and flavonoids. *F. regia* stood out for its exceptionally high content of total phenolics, while *D. chinensis* surpassed the other flowers in soluble sugar content. The aroma profile analysis revealed a diverse array of volatile organic compounds, with each species having its own unique composition. *C. moschata* was characterized by *p*-dimethoxybenzene and *D. chinensis* by non-terpene compounds; *F. regia* displayed high amounts of decanal and nonanal, while *V. cornuta* was rich in myrcene and α-farnesene. These findings provide valuable insights into the secondary metabolites and aroma profiles of these flowers, enhancing our understanding of their bioactive compounds and potential health benefits.

## 1. Introduction

The Mediterranean diet is widely recognized as the healthiest dietary pattern globally, mainly based on the consumption of vegetable foods sourced from the varied Mediterranean flora [1]. Ethnobotanical studies have provided detailed insights into the traditional use of edible flowers (EFs) in Mediterranean cuisine [2]. Indeed, floriphagia, the practice of consuming flowers as food, has been a cultural tradition in this region for centuries and continues to gain popularity in modern times [3]. Furthermore, scientific research conducted in recent years has supported and highlighted the nutritional and functional value of EFs [1,4,5]. EFs are known to contribute to the health-promoting properties of the human diet. They serve as excellent sources of essential macro- and micronutrients, thereby enhancing the overall nutritional value of dishes. Additionally, EFs provide a wide range of phytonutrients, including antioxidants, which play a crucial role in promoting human health [1,6]. 

*Cucurbita* spp. flowers (Cucurbitaceae family, common name: squash blossom) have a rich history of consumption in various parts of the world, including America, India, Europe, and Italy. Numerous documents [7,8] and traditional recipes, as well as the endorsement of renowned chefs, attest to their culinary significance. These plants exhibit a monoecious reproductive structure, producing separate staminate (male) and pistillate (female) flowers at the stem nodes [9]. The trumpet-shaped flowers, often displaying yellow or orange hues, should be harvested in the early morning since they bloom at dawn and wither by noon [9]. *Cucurbita* spp. flowers are widely appreciated for their versatility, ease of cooking, and delightful flavor [10]. While extensive research has been conducted on *C. pepo* and *C. maxima* flowers [11,12], those of *C. moschata* have received comparatively less attention. This particular species holds strong local ties to the Ligurian region of North Italy, where the cultivar ‘Tromboncino’ is commonly known as ‘zucchina trombetta’, which is named after the distinctive shape of the fruit. 

The cultivation of *Dianthus* spp. flowers (Caryophyllaceae family, common name: carnation) can be traced back to Ancient Greece. These flowers, highly regarded for their ornamental beauty, were even used as food flavoring in various culinary preparations [13]. To this day, this species continues to be utilized as an actual ingredient in several recipes [14]. The petals of *Dianthus* flowers are characterized by their distinctive bitter notes, which are enhanced at the petal basis [15]. While several species of *Dianthus* (e.g., *D. caryophyllus*, *D*. × *barbatus*, *D. chinensis*) are recognized as edible, the information regarding their nutritional features is limited, as most studies have primarily focused on their antioxidant properties [16].

*Fuchsia* spp. flowers (Onagraceae family) are predominantly cultivated for their vibrant color, and they are relatively less explored as EFs. The pendulous flowers of this genus exhibit a unique ear-drop shape, earning them the common name ‘lady’s eardrop’ [17]. This genus is intriguing as the flowers serve as sources of anthocyanins; the sepals of many species display bright red hues, while the petals can vary in color from white to dark red, pink, purple, blue and orange [17]. In some cases, even the pollen may be pigmented [18]. To the best of our knowledge, only *F.* × *hybrida* has been investigated as an EF so far [19], while very limited information is currently available on *Fuchsia regia*. 

*Viola* spp. flowers (Violaceae family, common name: viola) hold a significant place among the EFs that have a long-standing history of consumption and that, today, are still among the most appreciated and popular ones. These flowers are particularly appreciated for their vibrant colors (single or multi-colored flowers), diverse shapes (flowers with several diameters are available on the market), and pleasant taste (sweet and refreshing) with a velvety texture [20]. Among the *Viola* genus, *V.* × *wittrockiana* and *V. tricolor* have been extensively studied and recognized as functional foods [21,22]. However, there is still potential for exploring new *Viola* species to expand the range of recognized edible flowers and cater to consumers’ preferences.

In this contest, we focused on examining the phytochemical, nutritional and mineral content of *Cucurbita moschata* L., *Dianthus chinensis* L., *Fuchsia regia* (Vand. ex Vell.) Munz., and *Viola cornuta* L. flowers. Additionally, for the first time, we have determined their aroma profiles and quantified the water-soluble group B vitamins. The aim of this work is to provide pivotal new information on the nutraceutical features of these flowers, contributing to the existing literature and hence strengthening the currently available data. It is worth noting that, to the best of our knowledge, no previous research have been carried out on the flowers of *C. moschata* cv ‘Tromboncino’ and *F. regia*, making this study particularly valuable in advancing our knowledge in this area.

## 2. Material and Methods

### 2.1. Chemicals

All the salts and reagents used to quantify the antioxidant compounds (Folin–Ciocalteu reagent, sodium hydroxide, sodium carbonate, sodium nitrite, aluminum chloride, gallic acid, catechin, malvin chloride), antioxidant activity (2,2-diphenyl-1-picrylhydrazyl), macro- and microelement standard solution (*Trace*CERT^®^, 1000 ppm), and soluble vitamins (sodium acetate, takadiastase, vitamins B_1_, B_2_, B_3_, and B_9_, ascorbic acid, N-ethylmaleimide, DL-dithiothreitol, iron chloride) were purchased from Sigma-Aldrich (St. Louis, MO, USA). The perchloric acid, nitric acid, sulfuric acid, ethanol, methanol, chloroform, trichloroacetic acid, and phosphoric acid were purchased from VWR International LLC (Radnor, PA, USA).

### 2.2. Plant Material and Cultivation

**Figure 1:**
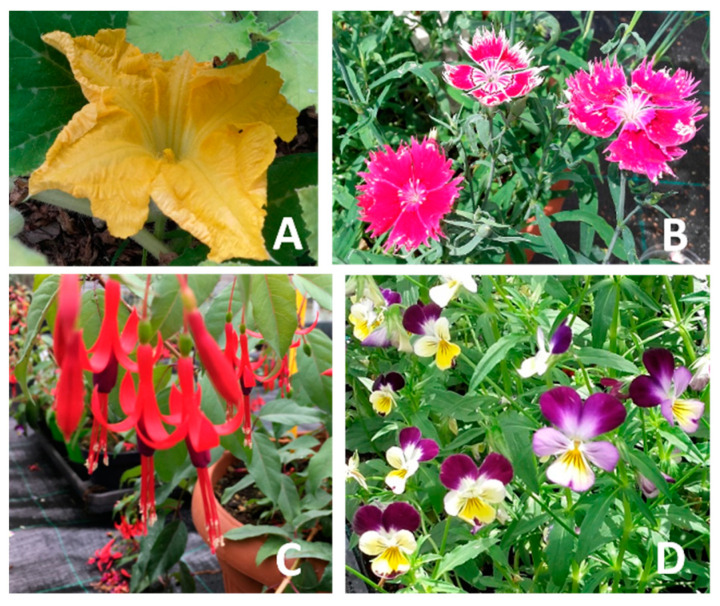
The four edible flowers: *Cucurbita moschata* Duchesne cv ‘Tromboncino’ (**A**), *Dianthus chinensis* L. (**B**), *Fuchsia regia* Vand. ex Vell. (**C**), and *Viola cornuta* L. (**D**).

*Dianthus chinensis* L., *Fuchsia regia* (Vand. ex Vell.) Munz., and *Viola cornuta* L. were cultivated in a greenhouse covered with an insect-proof net at the CREA Research Centre for Vegetable and Ornamental Crops (CREA, Sanremo, Imperia, Italy, GPS: 43.816887, 7.758900). On the other hand, *Cucurbita moschata* Duchesne cv ‘Tromboncino’ was grown in an open field near the greenhouses. The seeds of *D. chinensis* were purchased from Pagano Costantino & F.lli S.r.l. (Scafati, Salerno, Italy). They were sown in late winter (February–March) and then transplanted into pots (14 cm of diameter, 0.5 L). *F. regia* plants were provided by the Chambre d’Agriculture des Alpes-Maritimes (CREAM, Nice, France) and cultivated in pots (30 cm of diameter; 9 L). *V. cornuta* seeds (N. Sgaravatti & C. Sementi S.p.a., Laterina Pergine Valdarno, Arezzo, Italy) were sown in autumn (September–October) and later transplanted into pots (14 cm of diameter, 0.5 L). All the plants were grown following organic practices, as described by Najar et al. [23], and details regarding the substrate composition, fertilization treatment, frequency, and type of irrigation can be found in Marchioni et al. [24]. Fresh flowers of the four species were harvested at their full maturity during the summer of 2021, ensuring that only fully open and flawless flowers were selected (Figure 1). 

### 2.3. Biochemical Analyses

Fresh flowers were carefully collected in the early morning and divided into three homogeneous biological replicates. Half of each group was immediately frozen in liquid nitrogen and then stored at −80 °C until further analyses. The other half was subjected to vacuum freeze-drying (Labconco, Kansas City, MI, USA) for 48 h (condenser temperature: −50 °C). Both the fresh-frozen and freeze-dried flowers (moisture content: *C. moschata*: 92.05 ± 0.45; *D. chinensis*: 79.77 ± 1.94; *F. regia*: 83.87 ± 6.30; *V. cornuta*: 82.99 ± 1.25) were used for subsequent analyses according to the specific protocol requirements. All the spectrophotometric measurements were performed using an ultraviolet (UV)-1800 spectrophotometer (Shimadzu Corp., Kyoto, Japan). Unless stated otherwise, the three above-mentioned biological replicates were used for each analysis. 

#### 2.3.1. Soluble Sugars (D-Glucose, D-Fructose, Sucrose) Determination

To extract these primary metabolites, frozen flowers (0.1 g) were homogenized in 1.2 mL of 5.5% (*v*/*v*) perchloric acid (HClO_4_) following the extraction method reported by Tobias et al. [25]. The determination of D-glucose, D-fructose and sucrose was performed using a Sucrose/D-Fructose/D-Glucose Assay Kit (Megazyme International Ireland, Co. Wicklow, Ireland) following the manufacturer’s instructions. The results were expressed as milligrams of D-glucose, D-fructose and sucrose per gram of fresh weight (FW).

#### 2.3.2. Organic Nitrogen, Macro- and Microelement Quantification

The determination of the organic nitrogen content was performed using the Kjeldahl method [26] using 0.2 g of freeze-dried samples. Additionally, the same amount of samples was digested in a mixture of nitric–perchloric acids (HNO_3_:HClO_4_, 5:2 *v*/*v*) for 2 h at 220 °C [27]. The resulting digested samples were subsequently used for the macro- and microelement quantification. Phosphorous was measured by spectrophotometry using the molybdenum blue method [28], while the cations and microelements were determined by atomic absorption spectrophotometry (SpectrAA 240FS, Varian Australia Pty Ltd., Mulgrave, Australia). The absorbance measurements were performed at specific wavelengths (λ): 766.5 nm (K), 589.0 nm (Na), 422.7 nm (Ca), 285.2 nm (Mg), 324.8 nm (Cu), 279.5 nm (Mn), 248.3 nm (Fe), and 213.9 nm (Zn) [29]. The organic nitrogen, total crude proteins and macroelements were expressed as mg/g of dry weight (DW), while the microelements were reported as µg/g DW.

#### 2.3.3. Secondary Metabolites and Radical Scavenging Activity (DPPH Assay)

Frozen samples (0.2 g) were utilized to quantify the total polyphenols (Folin–Ciocalteu method), flavonoids, anthocyanins and carotenoids content following the procedures described by Marchioni et al. [30]. The results were expressed as mg gallic acid equivalents (GAEq)/g fresh weight (FW) (polyphenols), mg catechin equivalents (CEq)/g FW (flavonoids), mg malvin chloride equivalents (MEq)/g FW (anthocyanins), and µg/g FW (carotenoids). The radical scavenging activity was determined using the DPPH assay [31], and the results were reported as the IC_50_ (mg/mL).

#### 2.3.4. Determination of the Vitamin B_1_, B_2_, B_3_, B_9_, and C Content

All the B vitamins were extracted and quantified in the flower samples following the method outlined by Sami et al. [32], with some modifications. Briefly, 3.125 mL of 0.1 N sulfuric acid (H_2_SO_4_) (VWR, Radnor, PA, USA) were added to 0.25 g of each lyophilized flower powder. The samples were then incubated for 30 min at 121 °C and then cooled at room temperature. The pH was adjusted to 4.5 using 2.5 M sodium acetate (Sigma-Aldrich Co., St. Louis, MO, USA). Next, 6.25 mg of Takadiastase enzyme (Sigma-Aldrich Co.) was added to the samples, which were then kept overnight at 35 °C. Afterwards, the mixture was diluted with 6.25 mL of distilled water and filtered through a Whatman No. 4 filter followed by a 0.45 µm filter. A volume of 20 µL from each filtered sample was injected into the HPLC system. Chromatographic separation was achieved using an isocratic delivery mobile phase consisting of methanol (VWR) (solvent A) and 0.023 M phosphoric acid (H_3_PO_4_) (VWR) adjusted to pH = 3.54 (solvent B) (A/B 33/67) in a Waters 2487 HPLC Absorbance UV-Vis Detector (Marshall Scientific, Hampton, NH, USA). The HPLC system was equipped with a reverse-phase Luna column (5 µm C18(2) 100 Å, liquid chromatography column 150 × 4.6 mm). The flow rate was set at 0.5 mL/min, and the absorbance was monitored at 270 nm. Quantification of the vitamin B_1_, B_2_, B_3_, and B_9_ content (Sigma-Aldrich Co.) was performed by comparing the results to the relative standard curves. Standard stock solutions for the vitamins were prepared as previously described by Aslam et al. [33] and Marzougui et al. [34]. 

The total ascorbic acid amount was spectrophotometrically determined according to the method of Kampfenkel et al. [35], with the prior sample extraction as described by Degl’Innocenti et al. [36]. The data obtained were reported as mg/100 g FW.

### 2.4. Volatile Organic Compounds Analyses

The analysis of the aroma profile was performed using the solid-phase microextraction (SPME) of the whole flowers following the methodology reported in our previous work [23]. Briefly, 1 g of fresh flowers was placed in a 25 mL glass vial and enclosed for 30 min to allow for headspace collection. The enclosure was conducted at room temperature (21 °C). A fiber (100 μm polydimethylsiloxanes (PDMS)) (St Louis, MO, USA), previously conditioned according to the manufacturer’s instructions, was inserted into the vial to collect the volatile compounds. After 30 min, the fiber was immediately injected onto the gas chromatograph, which was set at a temperature 250 °C for thermal desorption of the analytes. The analyses were performed in duplicate for each species. Gas chromatography- electron impact mass spectrometry (GC-EIMS) was utilized to analyze the compounds desorbed from the SPME fiber, as reported in the previously cited work [23]. The separation of the compounds was achieved using an HP-5MS capillary column. 

### 2.5. Statistical Analysis

The biochemical results were subjected to statistical analysis using a one-way analysis of variance (ANOVA) in Past3 (version 3.15). Post hoc comparisons were performed using either the Tukey Honest Significant Difference (HSD) test or the Mann–Whitney U test, depending on the homogeneity of the variance, as determined by the Levene test. A significance of *p* < 0.05 (letters) was used as the cut-off for determining statistical significance. The biochemical and volatile compositions underwent comprehensive multivariate statistical analyses utilizing JMP software (SAS Institute Inc. JMP^®^. Version 16, SAS Institute Inc., 2021). To perform the Principal Component Analysis (PCA), a correlation matrix of the headspace complete VOC composition with a 40 × 4 matrix (40 individual compounds × 4 samples species) was utilized to determine the eigenvalues and eigenvectors. The resulting plot was generated by selecting the two principal components with the highest variance. Additionally, a two-way Hierarchical Cluster Analysis (HCA) was carried out using Ward’s method, and the squared Euclidean distances were used as a measure of similarity. These sophisticated analyses allowed for a deeper understanding of the relationships and patterns within the data.

## 3. Results and Discussion

Although extensive research has been conducted on the biological activity of EFs, there is still a lack of comprehensive nutritional information, even for some common species [13]. To address this issue, the present study aimed to quantify the primary metabolites, minerals, antioxidant compounds and water-soluble vitamins in the flowers of *Cucurbita moschata* cv ‘Tromboncino’, *Dianthus chinensis*, *Fuchsia regia*, and *Viola cornuta* flowers. Additionally, a particular emphasis was placed on the flowers’ aroma profile, recognizing its significance as a crucial characteristic of EFs that may influence consumers’ purchasing decisions [37].

### 3.1. Soluble Sugars, Total Crude Protein and Mineral Composition

A sweet taste is generally perceived as pleasant by our senses [38], and among the main compounds that activate human sweet receptors, there are soluble sugars such as glucose, fructose, and sucrose [39]. Among the four investigated species, *D. chinensis* showed the highest content of soluble sugars, followed by *F. regia*, *C. moschata* and *V. cornuta* (Table 1). 

In particular, D-glucose was consistently the most abundant sugar, while sucrose was absent from the *C. moschata* and *F. regia* flowers (Table 1). Previous studies reported the total soluble sugar content of EFs belonging to the same genera as the species included in this work [40,41,42], but only a few of them quantified D-glucose, D-fructose and sucrose separately. *V. cornuta* flowers are characterized by a lower amount of soluble sugars than white and red pansies (*Viola* × *wittrockiana* Gams), while the concentration of sucrose is similar to that found in yellow *Viola* × *wittrockiana* and in *V. cornuta* flowers [43]. In *D. chinensis* flowers, higher quantities of sucrose were detected in this work if compared to the work of Stefaniak and Grzeszczuk [41], and to the best of our knowledge, no papers have been published on the soluble sugar content of *Fuchsia* spp. until now.

The flowers’ crude protein content was indirectly measured by nitrogen determination, using a nitrogen-to-protein conversion factor (NPCF) of 6.25, as already reported by several authors [15,40,44]. The *C. moschata* and *V. cornuta* flowers showed the highest crude protein content, followed by *D. chinensis* and *F. regia* (Table 2).

In the *Cucurbita* genus, the content of this macronutrient appears to be influenced by the genotype of the plant, resulting in significant differences between species, cultivars and/or varieties. As regards *C. moschata*, the flowers’ crude protein content ranged from 181 to 318 mg/g DW, as reported by Bieżanowska-Kopeć et al. [42] and Toro-Vélez et al. [45]. Similar variability between flower varieties was observed in *C. maxima* Duchesne (149–222 mg/g DW) and *C. pepo* L. (219–266 mg/g FW) [42,44,45]. The flowers of the Ligurian cultivar (*C. moschata* cv. ‘Tromboncino’) evaluated here can only be compared to those of *C. maxima* var. Ambili (144 and 148 mg/g DW, respectively) [46]. On the contrary, the total crude protein content of *D. chinensis* flowers does not seem be affected by the variety [47], and our samples only slightly exceeded the amount of crude protein reported in *D. caryophyllus* L. [48]. The discrepancies in the crude protein content between *V. cornuta* and other *Viola* spp. fall within the range of 2–3% DW [22,49], while *F. regia* exhibits significantly higher levels of this macronutrient compared to *F.* × *hybrida* Voss flowers [50]. Despite the above-mentioned differences between the literature data and our results, at least three out of the four species here investigated could serve as a complementary protein source in the daily diet. Their content is higher than that of some of the well-known EFs, such as *Antirrhinum majus* L. (38 mg/g DW), *Centaurea cyanus* L. (69 mg/g DW), and *Tagetes erecta* L. (79 mg/g DW) [50,51], as well as of certain fruits (9–67 mg/g DW) [52]. However, it should be noted that while some Mexican EFs were reported to contain 251–274 mg/g DW of crude protein [44], our four species cannot be compared to some common legumes (240–261 mg/g DW), which are well known as a significant vegetable protein source [53]. 

Regarding minerals, calcium (Ca), potassium (K) and phosphorous (P) were found in the highest concentrations among the macroelements quantified in the four EFs, while iron (Fe), zinc (Zn) and manganese (Mn) were the micronutrients with the highest amount (Table 2). When comparing our results to the literature, we observed a significantly higher content of Ca, whereas the differences concerning K and P were much smaller and sometimes negligible. In more detail, Ca was quantified as 1.2–8.7 mg/g DW in *Cucurbita* spp. [42,45,46], 0.4–4.3 mg/g DW in *Dianthus* spp. [47,50], 2.9 mg/g DW in *F.* × *hybrida* [49], and 1.8–9.6 in *Viola* spp. flowers [50,54,55]. Correct plasma Ca homeostasis is essential for several vital functions in humans, such as maintaining skeletal health, regulating hormonal secretion, transmitting nerve impulses, and supporting vascular activities [56]. Particular attention should be paid to vegan and meat-oriented diets, as they have been associated with a reduction in Ca intake [57]. Considering that the daily recommended intake (DRI) of Ca is 800 mg [58], consuming around 14–16 dried *F. regia* flowers could provide around one-eighth of this amount (one flower = 70–80 mg DW, personal observation). On the contrary, the contribution of the four investigated EFs to reaching the DRI of K and P (2000 and 700 mg, respectively) [58] is very limited. However, *Agastache foeniculum* (Pursh) Kuntze flowers could be considered a potential source of K (96.9 mg/g DW), while to the best of our knowledge, *Monarda dydima* L. and *M. fistulosa* L. are the EFs with the highest P content (9.2 and 9.1 mg/g DW) [47].

As regards microelements, the amount of Fe was remarkably lower in our *C. moschata* cultivar than in other *Cucurbita* flowers [42,45,46]. In particular, *C. moschata* ‘Butternut’ and *C. maxima* ‘Atlantic Giant’ showed the highest content: 841 and 802 µg/g DW [42]. Significant differences were also observed between *V. cornuta* and *V. tricolor* flowers, since the latter were reported to contain 386 µg/g DW of Fe, around 6-fold more than our samples [55]. Despite these remarkable values reported in the literature, only the two noteworthy *Cucurbita* varieties may contribute significantly to the dietary Fe intake (DRI = 14 mg) [58], particularly when consumed as fresh flowers. In fact, a very large and unrealistic amount of *V. tricolor* (and especially of our four flowers) would be necessary to reach the DRI of Fe. No substantial differences were observed in the Mn levels between the investigated EFs and other flowers belonging to the same genera. However, the Zn content was significantly lower (with the exception of *D. chinensis*) [42,50,54]. Notably, these latter two microelements were particularly abundant in *Helicrysum italicum* subsp. Picardii Franco flowers (Mn: 216 µg/g DW; Zn: 310 µg/g DW) [59].

### 3.2. Antioxidant Compounds and Radical Scavenger Activity

EFs are also sources of a high variety of biologically active low molecular phytochemicals, including phenolic compounds and carotenoids. Polyphenols are one of the most numerous and widely distributed classes of plant secondary metabolites, essential for growth, pigmentation, reproduction, resistance to pathogens, and several other functions [60]. Concerning the four flowers studied in this work, the total polyphenolic content (TPC) was significantly higher in the flowers of *F. regia*, followed by the more limited quantities determined in *V. cornuta* > *D. chinensis* > *C. moschata* (Table 3). 

The differences in the total phenolic content between the *F. regia* and other *Fuchsia* spp. flowers (*F.* × *hybrida* and *F. magellanica* Lam.) were also remarkable (30.1 mg/g FW vs. 3.5 and 8.0 mg/g FW, respectively) [50,61]. This high TPC in *F. regia* is particularly noteworthy as it surpasses the values found in many other EFs, including some of those currently available on the market [12,62,63]. 

The TPC of *Dianthus*, *Viola*, and *Cucurbita* flowers has been more extensively studied, and variable results have been reported. For *D. chinensis*, the TPC values ranged from 12.3 to 5.3 to 1.2 mg/g FW in three independent studies [41,61,64]. Similar variability has been observed for *D. caryophyllus* and *D. barbatus* L. [50,61,65,66].

Regarding *V. cornuta* flowers, a comparable TPC was reported by Marchioni et al. [20], while our sample was characterized by a higher amount of polyphenols than *V.* × *wittrockiana* [22,50], *V. arvensis* Murray [67], and *V. alba* subsp. *dehnhardtii* (Ten.) W. Becker [68]. Lastly, no significant differences in the TPC were observed between our *C. moschata* samples and the *C. pepo* flowers investigated by Socha et al. [69], representing a single exception. In fact, several varieties of *C. maxima*, *C. moschata* and *C. pepo* showed a much greater amount of polyphenols (1–3.3 mg/g FW) [42,70,71].

The total flavonoid content (TFC) followed the same trend as the polyphenols, and the discrepancies between our results and the data reported in the literature were also confirmed for the former groups of metabolites [50,68]. 

The total anthocyanin content (TAC) is mainly represented in *F. regia* and *V. cornuta*, followed by the *D. chinensis* flowers. These pigments, which are primarily blue, red and purple, were not detected in *C. moschata*. This result agrees with previous findings that highlight the bright and varied colors of *Fuchsia* flowers, which are primarily attributed to anthocyanins [72]. These secondary metabolites also have a crucial role in *Viola* spp. flower pigmentation, as supported by molecular and biochemical investigations [73,74,75,76].

Carotenoids are another flower pigments responsible for yellow, orange, and red colors. In our study, the highest amount was quantified in *V. cornuta*, followed by *C. moschata* > *D. chinensis* > *F. regia* (Table 3). These findings, along with the data on flavonoids, suggest that *V. cornuta* flowers have a richer profile of both metabolites compared to the others. This is due to their peculiar color patterning (Figure 1), where pale, yellow and purple areas coexist, each of which is determined by the relative amount of several pigments [77,78]. Moreover, when compared to the literature data, our *V. cornuta* samples showed a higher carotenoids content than *V. tricolor* and bicolored *V.* × *wittrockiana* flowers [39,42]. On the contrary, *C. moschata*, *D. chinensis* and *F. regia* showed a lower carotenoids concentration than other flowers belonging to the same genera [41,42,61].

The flowers’ radical scavenging activity (DPPH assay), expressed as the half-maximal inhibitory concentration (IC_50_), was consistent with the content of secondary metabolites: the higher they are, the lower the IC_50_ values are (and hence the higher the respective antioxidant activity) (Table 3). Similar values have been observed in several edible flowers belonging to the Lamiaceae family [24].

### 3.3. Water-Soluble Vitamins (B_1_, B_2_, B_3_, B_9_, C)

Water-soluble vitamins (WSVs) are a group of structurally and functionally unrelated compounds that share the characteristics of being essential for normal cellular functions, growth, and development [79]. Since humans cannot synthesize these vitamins, their dietary intake is pivotal, and deficiencies can lead to serious disorders, including well-known pathologies such as beriberi (thiamine, vitamin B_1_), pellagra (niacin, vitamin B_3_), anemia (pyridoxine, vitamin B_6_), and scurvy (ascorbic acid, vitamin C) [80]. In most developed countries, people rarely take inadequate amounts of WSVs, even if some deficiencies have been observed in vegan diets [80,81]. However, several plant-based sources are characterized by a good amount of vitamins B and C (e.g., whole grain-based foods, legumes, green leafy vegetables, nuts, and fruits), even if very limited information is available for EFs. In this work, vitamins B_1_, B_2_, B_3_ and folic acid (vitamin B_9_) were quantified for the first time in *C. moschata*, *D. chinensis*, *F. regia* and *V. cornuta* flowers, while a few other authors investigated the vitamin C content in species belonging to the same genera.

Among the flowers studied, *C. moschata* had the highest content of vitamin B, with the exception of riboflavin (B_2_) (Table 4), which was poorly represented in all the species (DRI = 1.4 mg) [82].

This flower could help to meet the DRI of these vitamins B_3_, B_1_, and B_9_ (16, 1.1 and 0.2 mg, respectively) [82], especially when consumed fresh. These findings are particularly significant, especially if compared to *Cucurbita* fruits, which are more commonly consumed than flowers, which contain a lower amount of thiamin (0.05 mg), niacin (0.49 mg), and total folate (0.03 mg per 100 g of raw edible portion with skin) [83]. However, it is important to consider that *Cucurbita* flowers are mostly consumed cooked, often boiled or grilled [10]. For these reasons, it is possible that some vitamins, such as thiamin, may undergo degradation due to the thermal effect of cooking [84]. Future studies should investigate the potential alteration in EFs’ vitamin content during the cooking process. To the best of our knowledge, only carotene, lycopene and polyphenols have been evaluated in cooked banana (*Musa* × *paradisiaca* L.) and neem (*Azadirachta indica* A. Juss.) flowers using different cooking methods [85].

All the flowers studied in this work showed higher vitamin B content than others reported in the literature, except for riboflavin. The thiamin content ranged between 0.03 and 1.05 mg/g DW, respectively, in *Celosia cristata* L. and *Bombax buonopozense* P. Beauv. flowers [86,87]. *M. paradisiaca* flowers were characterized by the lowest amount of vitamin B_3_ (0.005 mg/100 g DW), while the highest content was reported in *Madhuca indica* J. F. Gmel (4.8 mg/100 g DW) [87,88]. Folic acid was generally very low (0.003–0.092 mg/100 DW) [86,89], even if an exceptional amount was detected in *Carica papaya* L. flowers (510 mg/100 g DW). As regards riboflavin, *M. indica* and *B. buonopozense* had the highest content of this vitamin (0.87 and 0.63 mg/100 g DW, respectively), while *C. cristata* the lowest (0.13 mg/100 g DW) [86,90]. 

Compared to the B vitamins, ascorbic acid has received greater interest. Our results revealed that *V. cornuta* had the highest concentration of vitamin C, followed by *D. chinensis* > *F. regia* > *C. moschata* flowers (Table 4). Concerning *Viola* spp. flowers, contradictory results have been published concerning *V. tricolor*, for which Ombra et al. [91] quantified 29 mg/100 g FW of ascorbic acid, while Grzeszczuk et al. [40] reported a concentration of 182 mg/100 g FW. A significantly lower amount was detected in our samples than in *D. chinensis* ‘Chianti’ (89 mg/100 g FW) and *C. pepo* flowers (17 mg/100 g FW) [41,70]. No information is available on *F. regia* flowers; hence, their ascorbic acid content was determined here for the first time. Vitamin C has received special attention in postharvest studies since this metabolite can decrease progressively over time [70,92]. This nutrient should reach consumers unaltered since it is involved in several human metabolic processes and shows remarkable antioxidant activity [81]. To achieve this, EFs should be consumed as soon as possible after their harvest, or suitable storage conditions need to be defined for each specific flower [93].

### 3.4. Volatile Organic Compound Analyses

The volatile organic compositions of the studied flower species are summarized in Table 5. 

A total of 40 chemical compounds were identified, with the identification rate ranging from 95.3% in *C. moschata* to 100% in both *D. chinensis* and *F. regia*. The predominant class of compounds in the aroma profile of *C. moschata* was uniquely represented by p-dimethoxybenzene (77.5%), which is a non-terpene derivative. To account for 94% of the total identified fraction, benzyl nitrile and 2-nitroethyl benzene were added in almost equal percentages (6.2% and 6.0%, respectively) to the main constituent. The presence of p-dimethoxybenzene in significant amounts has previously been reported in the nectar of male flowers of *C. pepo* [94,95], and it has been identified as the most attractive compound to specialist squash bee pollinators [96]. Interestingly, the HS-GC-MS analysis of *C. moschata* fruit reported by [97] revealed 3-methyl butanal to be almost the only compound, with benzaldehyde also present in the flower (0.7%) sample. In a separate study by Zhao et al. [98], tridecane, nonanal, myrcene and octanal were found in the leaf aroma profile of this species, but only nonanal was observed in the studied flowers.

*D. chinensis* exhibited a similar pattern to *C. moschata* in terms of the prevalence of non-terpene compounds (NTs), which accounted for 60.6% of the floral volatiles. These non-terpene compounds were predominantly ester (32.5) and aldehyde (25.7%). Notably, cis-hexenyl acetate (31.2%) and decanal (16.6%) were the major constituents, comprising over 45% of the floral volatiles. Another intriguing finding was the presence of a high amount of oxygenated sesquiterpenes (OSs), particularly dihydro-β-agarofuran (29.7%). A similar abundance of cis-hexenyl acetate (27.1%) was reported in the floral scent of *D. deltoides* L. [99]. It is worth mentioning that the prevalence of non-terpene was also observed in the volatile fraction of *D. carmelitarum* Reut. ex Boiss., where tricosane (21.8%) followed by α-thujene (20.5%) and undecanal (18.6) were the major compounds [100]. Interestingly, none of these compounds were detected in the studied species. 

Decanal was shown to be the major constituent (29.8%) of *F. regia*, followed by another aldehyde, nonanal (19.0%). This species exhibited a relatively equal distribution of three classes of volatiles: OSs, oxygenated diterpenes (ODs), and apocarotenoids (ACs). Each class was exclusively represented by one compound: (E,E)-farnesyl acetate (14.0%) for OSs, trans-geranylgeraniol (10.3%) for ODs, and cis-geranyl acetone (12.1%) for ACs, respectively. The majority of these compounds are known for their sweet and floral notes, as indicated in Table 6. 

It is worth noting there are no previous reports of the spontaneous release of these compounds in *F. regia* or the *Fuchsia* genus as far as the authors are aware.

Monoterpene hydrocarbons (40.6%) were the main class of floral spontaneous emission in *V. cornuta*, followed by sesquiterpene hydrocarbons (SHs, 35.2%). In detail, this species was characterized by myrcene (36.7%) and α-farnesene (34.5%). These compounds likely contribute to the green and floral notes observed in horned pansies (Table 6). On the contrary, in other *Viola* spp., such as *V. calcarata* L. and *V. dubyana* Burnat ex Gremli, methyl salicylate was reported as the major constituent in the EO analyzed by HS-SPME [114], while in *V. inconspicua* Blume flowers, alkanes, aldehydes, and ketones were the main volatile compounds, with *n*-heneicosane (25.85%) being the predominant compound [115]. 

### 3.5. Multivariate Statistical Analyses

**Figure 2:**
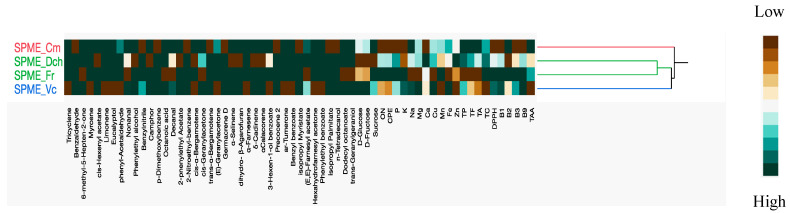
Multivariate statistical analysis using two-way Hierarchical Cluster Analysis (HCA).

Principal Component Analysis (PCA) and Hierarchical Cluster Analysis (HCA) were performed on the volatilomic data matrix, along with the soluble sugars, total crude protein, mineral composition, and water-soluble vitamins. The two-way HCA analysis (Figure 2) revealed three primary groups (A (red), B (green), and C (blue)). 

Group B demonstrated a close clustering of *D. chinensis* and *F. regia* emissions. In contrast, the other two groups (A and C) were distinctly characterized by individual sample species (A: *V. cornuta*; C: *C. moschata*).

**Figure 3:**
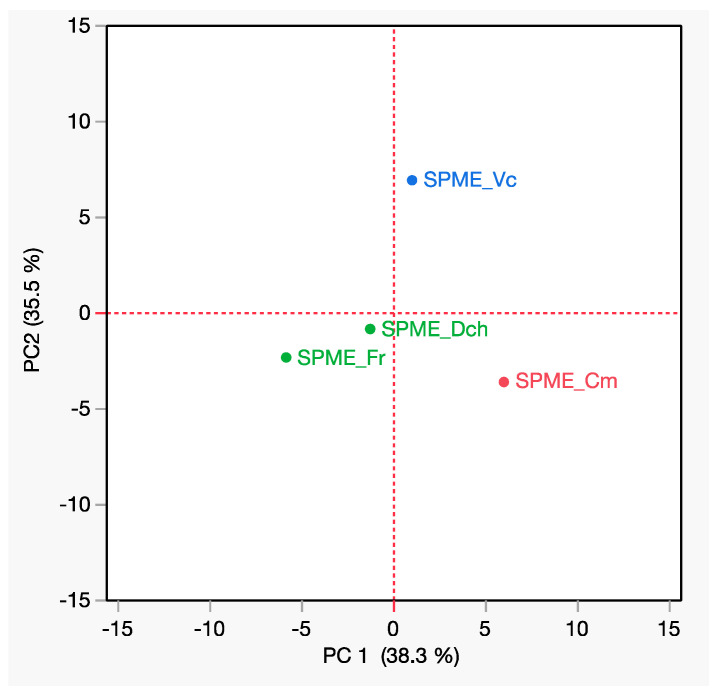
PCA score plot of the biochemical and volatile compositions of the four EFs.

In the PCA analysis, as depicted in Figure 3, the first two principal components, PC1 and PC2, collectively accounted for 73.8% of the variance.

Notably, PC1 was instrumental in distinguishing the emissions of *V. cornuta* and *C. moschata* (both showing positive values along PC1) from the emissions of the other studied species. Meanwhile, PC2 played a vital role in differentiating between *V. cornuta* and *C. moschata*, with *V. cornuta* located in the upper right quadrant (positive on both PC1 and PC2) and *C. moschata* positioned in the lower right quadrant (positive on PC1 and negative on PC2). The two other studied species were situated in the lower left region.

**Figure 4:**
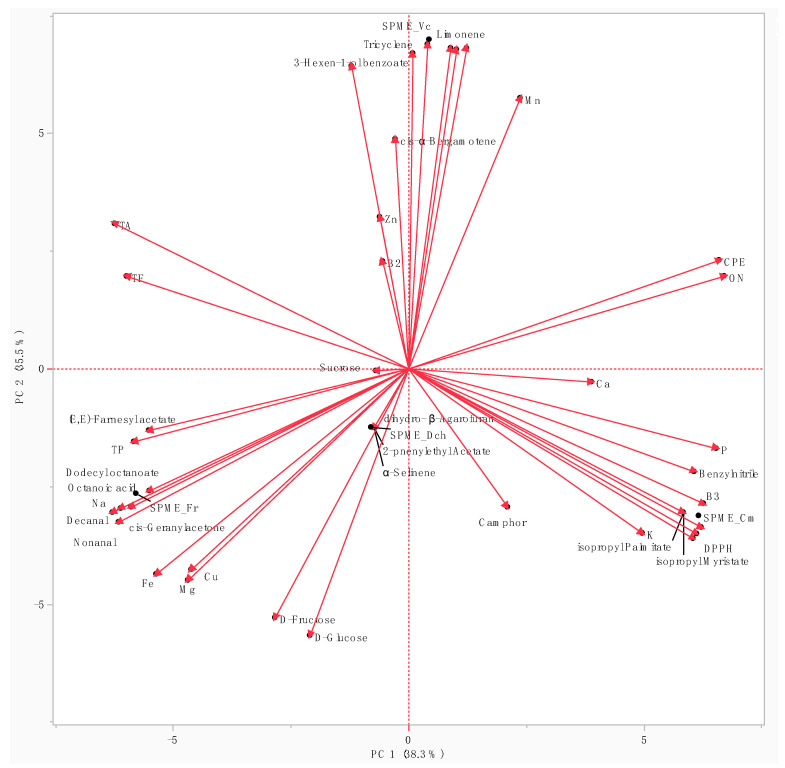
PCA biplot of the biochemical and volatile compositions of the four EFs.

The biplot of the PCA (Figure 4) analysis highlights *V. cornuta*’s distinct position, influenced by the presence of unique compounds such as tricyclene (1), limonene (6), and 3-hexen-1-ol benzoate (29) in its flower emission.

Additionally, *V. cornuta* displayed higher manganese and zinc content compared to the other species. Conversely, *C. moschata*’s position in the biplot was attributed to its richness in vitamin B_3_ and phosphorus, and to its scarce antioxidant activity (the higher the IC_50_ value, the lower the radical scavenging activity).

Both *D. chinensis* and *F. regia* were characterized by the presence of decanal (16) and cis-geranyl acetone (20), which were exclusive to these species. Additionally, they exhibited higher levels of iron, sodium, D-glucose, and D-fructose, explaining their close clustering and their positioning in the same left quadrant (negative along both PC1 and PC2). 

## 4. Conclusions

This study provides valuable insight into the composition of the bioactive compounds, antioxidant activity, and aroma profiles of the investigated edible flowers. These findings not only enhance our understanding of the potential health benefits associated with these flowers but also unlock opportunities for their utilization in functional foods, natural remedies, and fragrance-related applications. However, further research is recommended to deepen our knowledge of these compounds, improve their shelf life and stability, and conduct sensory evaluation.

## Figures and Tables

**Table 1:** Determination of the soluble sugars (D-glucose, D-fructose, sucrose) of the four edible flowers. Data are expressed as the mean ± standard error (*n* = 3).

Species/Varieties				
	*C. moschata*	*D. chinensis*	*F. regia*	*V. cornuta*
D-glucose (mg/g FW)	3.73 ± 0.35 ^b^	5.78 ± 0.10 ^a^	4.73 ± 0.45 ^ab^	1.26 ± 0.10 ^c^
D-fructose (mg/g FW)	2.44 ± 0.14 ^c^	4.74 ± 0.13 ^a^	3.77 ± 0.28 ^b^	0.62 ± 0.05 ^d^
Sucrose (mg/g FW)	ND ^c^	1.36 ± 0.04 ^a^	ND ^c^	0.33 ± 0.02 ^b^

^(a–d)^ Letters indicate, for each quantification, significant differences using a one-way ANOVA with the Tukey HSD or the Mann–Whitney U tests, with a cut-off significance of *p* < 0.05. Abbreviations: FW = fresh weight; ND = not determined.

**Table 2:** Determination of the organic nitrogen, macro- and microelement content of the four edible flowers. Data are expressed as the mean ± standard error (*n* = 3).

Species/Varieties				
	*C. moschata*	*D. chinensis*	*F. regia*	*V. cornuta*
Organic nitrogen (mg/g DW)	23.15 ± 1.82 ^a^	15.50 ± 0.03 ^b^	9.75 ± 0.43 ^c^	20.07 ± 0.02 ^ab^
Crude protein estimation (mg/g DW)	144.69 ± 11.37 ^a^	96.88 ± 0.00 ^b^	60.94 ± 2.71 ^c^	129.41 ± 0.10 ^ab^
Phosphorous (mg/g DW)	5.95 ± 0.08 ^a^	3.94 ± 0.02 ^b^	3.55 ± 0.05 ^c^	4.18 ± 0.08 ^b^
Potassium (mg/g DW)	31.54 ± 0.76 ^a^	30.91 ± 0.45 ^a^	26.11 ± 0.54 ^b^	26.49 ± 0.73 ^b^
Sodium (mg/g DW)	0.15 ± 0.01 ^c^	0.67 ± 0.03 ^b^	2.07 ± 0.02 ^a^	0.24 ± 0.00 ^c^
Calcium (mg/g DW)	47.51 ± 2.20 ^b^	46.63 ± 2.13 ^b^	90.54 ± 1.65 ^a^	29.70 ± 1.02 ^c^
Magnesium (mg/g DW)	4.98 ± 0.05 ^a^	2.48 ± 0.11 ^d^	3.75 ± 0.03 ^c^	4.04 ± 0.03 ^b^
Copper (µg/g DW)	11.86 ± 0.48 ^b^	11.23 ± 0.42 ^bc^	18.83 ± 0.91 ^a^	9.24 ± 0.26 ^c^
Manganese (µg/g DW)	48.15 ± 1.26 ^c^	54.91 ± 0.85 ^b^	40.51 ± 1.04 ^d^	61.59 ± 1.28 ^a^
Iron (µg/g DW)	66.84 ± 0.37 ^b^	73.33 ± 2.41 ^ab^	89.24 ± 4.60 ^a^	61.00 ± 4.82 ^b^
Zinc (µg/g DW)	67.42 ± 1.60 ^a^	51.60 ± 0.33 ^b^	72.72 ± 2.35 ^a^	76.92 ± 0.86 ^a^

^(a–d)^ Letters indicate, for each quantification, significant differences using a one-way ANOVA with the Tukey HSD or the Mann–Whitney U tests, with a cut-off significance of *p* < 0.05. Abbreviations: DW = dry weight.

**Table 3:** Determination of the total polyphenols, flavonoids, anthocyanins, and carotenoids in the four edible flowers, and their radical scavenger activity (DPPH assay). Data are expressed as the mean ± standard error (*n* = 3).

Species/Varieties				
	*C. moschata*	*D. chinensis*	*F. regia*	*V. cornuta*
Total polyphenols (mg GAEq/g FW)	0.67 ± 0.04 ^d^	2.05 ± 0.19 ^c^	30.06 ± 0.78 ^a^	5.79 ± 0.25 ^b^
Total flavonoids (mg CEq/g FW)	0.30 ± 0.01 ^d^	1.06 ± 0.08 ^c^	4.19 ± 0.17 ^a^	2.89 ± 0.17 ^b^
Total anthocyanins (mg MEq/g FW)	ND ^c^	0.65 ± 0.02 ^b^	1.36 ± 0.06 ^a^	1.20 ± 0.06 ^a^
Total carotenoids (µg/g FW)	13.85 ± 0.31 ^b^	7.45 ± 0.61 ^bc^	0.78 ± 0.05 ^c^	95.35 ± 5.57 ^a^
Antiradical activity (DPPH) (IC_50_ mg/mL)	11.16 ± 0.31 ^d^	4.24 ± 0.23 ^c^	0.80 ± 0.00 ^a^	1.52 ± 0.08 ^b^

^(a–d)^ Letters indicate, for each quantification, significant differences using a one-way ANOVA with the Tukey HSD or the Mann–Whitney U tests, with a cut-off significance of *p* < 0.05. Abbreviations: FW = fresh weight; GAEq = gallic acid equivalents; CEq = catechin equivalents; MEq = malvin equivalents; DPPH = 2,2-diphenyl-1-picrylhydrazyl.

**Table 4:** Determination of the water-soluble B vitamin and total ascorbic acid content of the four edible flowers. Data are expressed as the mean ± standard deviation (*n* = 2) (B vitamins) or mean ± standard error (*n* = 3) (total ascorbic acid).

Species/Varieties				
	*C. moschata*	*D. chinensis*	*F. regia*	*V. cornuta*
Thiamine (B_1_) (mg/100 g DW)	13.63 ± 0.34 ^a^	4.98 ± 0.10 ^b^	2.13 ± 0.00 ^c^	2.35 ± 0.01 ^c^
Riboflavin (B_2_) (mg/100 g DW)	0.03 ± 0.00 ^c^	0.16 ± 0.01 ^a^	ND ^d^	0.11 ± 0.00 ^b^
Nicotinamide (B_3_) (mg/100 g DW)	218.90 ± 0.76 ^a^	169.69 ± 1.87 ^b^	107.70 ± 1.73 ^d^	128.89 ± 2.22 ^c^
Folic acid (B_9_) (mg/100 g DW)	13.13 ± 1.18 ^a^	3.16 ± 0.16 ^b^	0.43 ± 0.06 ^c^	0.40 ± 0.01 ^c^
Total ascorbic acid (mg/100 g FW)	0.85 ± 0.05 ^d^	24.70 ± 1.34 ^b^	8.90 ± 0.40 ^c^	42.55 ± 1.69 ^a^

^(a–d)^ Letters indicate, for each quantification, significant differences using a one-way ANOVA with the Tukey HSD or the Mann–Whitney U tests, with a cut-off significance of *p* < 0.05. Abbreviations: FW = fresh weight; DW = dry weight; ND = not determined.

**Table 5:** Volatile organic compounds in the studied species using SPME-GC-IEMS (Cm: *Cucurbita moschata*, Dch: *Dianthus chinensis*, Fr: *Fuchsia regia*; Vc: *Viola cornuta*).

N°	Compounds		Class	LRI^exp^	LRI^lit^	SPME_Cm	SPME_Dch	SPME_Fr	SPME_Vc
						*Relative abundance (%)*			
1	Tricyclene		Mh	928	924	-	-	-	1.4 ± 0.23
2	Benzaldehyde	ADH	Nt	962	966	0.7 ± 0.04	-	-	-
3	6-methyl-5-hepten-2-one	KET	Nt	987	988	-	-	4.1 ± 1.35	-
4	Myrcene		Mh	993	987	-	-	-	36.7 ± 1.58
5	cis-hexenyl acetate	EST	Nt	1008	1006	-	31.2 ± 1.05	-	-
6	Limonene		Mh	1030	1028	-	-	-	2.5 ± 0.26
7	Eucalyptol		Om	1032	1033	-	-	-	2.0 ± 0.03
8	Phenylacetaldehyde	ADH	Nt	1045	1049	0.4 ± 0.12	-	-	5.0 ± 0.28
10	Nonanal	ADH	Nt	1102	1102	0.7 ± 0.19	8.3 ± 0.49	19.0 ± 2.78	0.8 ± 0.28
11	Phenylethyl alcohol	ACH	Nt	1111	1112	-	2.4 ± 0.51	-	-
12	Benzylnitrile	OTR	Nt	1138	1143	6.2 ± 0.74	-	-	0.8 ± 0.03
13	Camphor		Om	1145	1146		2.4 ± 0.90	-	-
14	*p*-dimethoxybenzene	ETR	Nt	1168	1165	77.5 ± 3.88	-	-	-
15	Octanoic acid	ACI	Nt	1183	1191	-	-	6.2 ± 0.84	-
16	Decanal	ADH	Nt	1204	1200	0.8 ± 0.47	14.6 ± 1.75	29.8 ± 2.19	2.3 ± 0.18
17	2-pnenylethyl acetate	ADH	Nt	1258	1256	-	2.8 ± 0.77	-	-
18	2-nitroethyl-benzene	OTR	Nt	1304	1306	6.0 ± 0.15	-	-	-
19	cis-α-bergamotene		Sh	1416	1415	-	0.7 ± 0.06	-	0.7 ± 0.12
20	cis-geranylacetone		Ac	1435	1434	-	2.1 ± 0.24	12.1 ± 0.25	-
21	trans-α-bergamotene		Sh	1438	1441	0.1 ± 0.03	-	-	-
22	(*E*)-geranylacetone		Ac	1456	1454	0.1 ± 0.02	-	-	1.0 ± 0.58
23	Germacrene D		Sh	1482	1485	0.3 ± 0.06	-	-	-
24	α-selinene		Sh	1495	1494	-	0.3 ± 0.07	-	-
25	Dihydro- β-agarofuran		Os	1496	1501	-	29.7 ± 2.68	-	-
26	α-farnesene		Sh	1508	1507	-	-	-	34.5 ± 4.52
27	δ-cadinene		Sh	1524	1519	-	4.0 ± 0.05	-	-
28	α-calacorene		Sh	1543	1542	-	0.2 ± 0.04	-	-
29	3-hexen-1-ol benzoate	BZD	Nt	1585	1585	-	1.3 ± 0.19	-	3.0 ± 0.45
30	Precocene 2	Chromene	Nt	1658	1656	0.3 ± 0.07	-	-	-
31	Ar-turmerone		Os	1664	1664	-	-	-	1.0 ± 0.09
32	Benzyl benzoate	BZD	Nt	1762	1676	-	-	-	2.1 ± 0.71
33	Isopropyl myristate		Os	1827	1759	0.4 ± 0.04	-	-	-
34	(*E,E*)-farnesyl acetate		Os	1843	1831	0.7 ± 0.04	-	14.0 ± 0.55	3.1 ± 0.11
35	Hexahydrofarnesyl acetone		Os	1844	1840	-	-	-	0.9 ± 0.07
36	Phenylethyl benzoate	EST	Nt	1853	1847	-	-	-	1.9 ± 0.43
37	Isopropyl palmitate	EST	Nt	1981	1859	0.3 ± 0.05	-	-	-
38	N-tetradecanol	ACH	Nt	2056	1999	0.8 ± 0.08	-	-	-
39	Dodecyl octanoate	EST	Nt	2160	2160	-	-	4.5 ± 0.49	-
40	trans-geranylgeraniol		Od	2201	2201	-	-	10.3 ± 0.26	-
Class of compounds						SPME_Cm	SPME_Dch	SPME_Fr	SPME_Vc
Monoterpene hydrocarbons (MHs)						-	-	-	40.6 ± 2.61
Oxygenated monoterpenes (OMs)						-	2.4 ± 0.90	-	2.0 ± 0.03
Sesquiterpene hydrocarbons (SHs)						0.4 ± 0.04	5.2 ± 0.05	-	-
Oxygenated sesquiterpenes (OSs)						1.1 ± 0.14	29.7 ± 2.68	14.0 ± 0.55	5.0 ± 0.09
Oxygenated diterpenes (ODs)						-	-	10.3 ± 0.26	-
Apocarotenoids (ACs)						0.1 ± 0.02	2.1 ± 0.24	12.1 ± 0.25	1.0 ± 0.58
Total non-terpene derivatives (NTs)						93.7 ± 1.40	60.6 ± 0.80	63.6 ± 1.53	15.9 ± 1.91
Non-terpene derivative		Alcohol (ACH)				0.8 ± 0.08	2.4 ± 0.51	-	-
		Acids (ACI)				-	-	6.2 ± 0.84	-
		Aldehydes (ADH)				2.6 ± 0.28	25.7 ± 2.57	48.8 ± 3.09	8.1 ± 0.58
		Esters (EST)				0.3 ± 0.05	32.5 ± 1.22	4.5 ± 0.49	7.0 ± 0.89
		Eters (ETR)				77.5 ± 3.88	-	-	-
		Ketones (KET)				-	-	4.1 ± 1.35	
		Chromene				0.3 ± 0.07	-	-	-
		others				12.2 ± 0.99	-	-	0.8 ± 0.03
	Total identified					95.3 ± 0.24	100 ± 0.00	100 ± 0.00	99.7 ± 0.43

LRI^exp^: Linear retention indices on a DB5 capillary column. LRI^lit^: Linear retention indices reported in NIST (NIST Chemistry WebBook, SRD 69) visited: 10th of March (03) 2024.

**Table 6:** Selection of aromatic compounds (>5%) for each flower and their corresponding odor characteristics as described in the literature.

Flower	Volatile Compound	Odor Description	References
*C. moschata*	*p*-dimethoxybenzene	Sweet, green, hay	[101]
	Benzylnitrile	Bitter almonds, spicy	[102]
	2-nitroethyl-benzene	Sweet, floral, spicy	[103]
*D. chinensis*	cis-hexenyl acetate	Green	[104]
	Dihydro- β-agarofuran	-	
	Decanal	Sweet, aldehydic, fatty, citrusy	[105,106]
	Nonanal	Candle-like, sweet orange-like, fatty and floral	[107]
*F. regia*	Decanal	Sweet, aldehydic, fatty, citrusy	[105,106]
	Nonanal	Candle-like, sweet orange-like, fatty, floral	[107]
	(*E*,*E*)-farnesyl acetate	Woody, floral	[108]
	Cis-geranylacetone	Magnolia, green, floral	[109]
	Trans-geranylgeraniol	-	
	Octanoic acid	Waxy, soapy	[110]
*V. cornuta*	Myrcene	Resinous, herbaceous, balsamic, geranium-like	[111]
	α-farnesene	Fruity, herbaceous	[112]
	Phenylacetaldehyde	Floral, lilac	[113]

## Data Availability

The original contributions presented in the study are included in the article, further inquiries can be directed to the corresponding author.
